# Household

**Published:** 1889-08

**Authors:** 


### HOUSEHOLD.

STRAWBERRY DUMPLINGS.—Put one pint ot sifted flour into a bowl, rub into it two ounces of butter, add a teaspoonful of salt, a heaping taaspoonful of baking powder and sufficient milk to moisten, about one gill, mix quickly, take out on a board and roll out into a sheet a quarter of an inch in thickness, cut into cakes with a round buscuit cutter, put about three strawberries in each cake, fold them over neatly and steam about twenty minutes. While they are steaming make the strawberry sauce.

STRAWBERRY SAUCE.—Beat two ounces of butter to a cream, adding gradually four ounces or a half cup of powdered sugar, then add twelve strawberries, one at a time, mashing and beating until the whole is perfectly light. If it has a separated or curdled appearance, add a little more sugar and stand in a cold place until wanted.

STRAWBERRY TARTS.—Roll out the paste into a thin sheet, then, with a round tin cutter, cut into cakes. Select a smaller cutter, and cut out the centres of two in every three round cakes, thus leaving one solid bottom with two rings placed on top of it to form the patty. Now, put these patties in the ice chest a half hour to cool, then bake in a moderately quick oven about thirty minutes or until a nice brown ; then, with a spoon, take out any unbaked portion that may remain in the centre. Brush the tops over with the unbeaten white of an egg, dust thickly with granulated sugar, return to the oven a few minutes to glaze. When ready to serve, fill with fresh strawberries, heap whipped cream over the top, and dust lightly with powdered sugar.

ANOTHER NEW VEGETABLE.—Another new vegetable has been introduced into France by M. Paillieux, the indefatigable collector of new alimentary plants. The plant has been received through the aid of M. Bouley, head gardener to the Maharajah of Cashmere. It is called the Congalou. This vegetable is a sort of turnip with the form of a radish, and with the skin of an attractive bright red color. The flavor is nearly that of the ordinary turnip, but very much stronger, the consistency of the root is such that it does not soften in cooking. It appears that in the Himalayan regions the Congalou is eaten as a salad, sliced in very thin rounds and highly seasoned.—*Vick's Magazine* for June.

TO REMOVE INK STAINS.—Ink made with nutgalls and copperas can be removed by using a moderately concentrated solution of oxalic acid, followed by use of pure water, and frequent drying with clean blotting paper. Most other black inks are erased by use of a weak solution of chlorinated lime, followed by dilute acetic acid and water, drying with blotters. Malachite green ink is bleached by aqua ammonia ; silver inks, by potassium cyanide or sodium hyposulphite. Some aniline colors are easily removed by alcohol, and nearly all by chlorinated lime followed by dilute acetic acid or vinegar. All these remarks apply to goods. The removal of such stains from tinted papers or colored dress goods is nearly impossible, in many cases, without impairing the color of the fabrics; and silk and woolen goods are liable to be acted upon by the chemicals so as to be seriously injured. In all cases apply the substances with camel's hair brushes or feathers, and allow them to remain no longer than is necessary, after which rinse well with water and dry with blotting papers. There is no reliable method for the removal of printing ink.

